# Long‐Term Glycemic and Metabolic Profiles Observed During Imeglimin Therapy in Japanese Patients With Type 2 Diabetes Mellitus

**DOI:** 10.1002/edm2.70263

**Published:** 2026-07-06

**Authors:** Yohei Fujita, Shuji Suganami, Yuka Morita, Toshio Matsui, Masahisa Hata, Masahiro Hatazaki

**Affiliations:** ^1^ Department of Diabetes and Endocrinology Osaka General Medical Center Osaka Osaka Japan

**Keywords:** C‐peptide index, glycated albumin, glycemic control, imeglimin, real‐world observational study, type 2 diabetes mellitus

## Abstract

**Aims/Introduction:**

Imeglimin is a first‐in‐class oral antidiabetic agent that improves glycemia through dual actions on insulin secretion and sensitivity. However, real‐world data on its long‐term metabolic effects, particularly on endogenous insulin secretion, remain limited. We aimed to evaluate 12‐month longitudinal changes in glycemic indices, an insulin secretion index, and treatment intensity during imeglimin therapy in routine clinical practice.

**Materials and Methods:**

This retrospective observational study included 73 Japanese patients with type 2 diabetes mellitus who continued imeglimin therapy for approximately 12 months. Hemoglobin A1c (HbA1c) and glycated albumin (GA) were assessed at baseline and follow‐up. Fasting plasma glucose and C‐peptide levels obtained between 10 and 14 months after initiation were used to calculate the fasting C‐peptide index (fCPI). Overall glucose‐lowering treatment intensity was quantified using the medication effect score (MES).

**Results:**

HbA1c significantly decreased from 8.55% ± 1.38% to 7.67% ± 0.99% over 12 months (*p* < 0.0001). GA also declined from the early phase after initiation, whereas the GA/HbA1c ratio showed only transient change without sustained differences. The fCPI increased from 1.14 [0.65–1.84] to 1.21 [0.79–2.01] (*n* = 69, *p* = 0.003), and an early increase was observed within 1–2 months. Despite frequent adjustments to background therapies, treatment intensity as assessed by MES did not change significantly.

**Conclusions:**

In a real‐world setting, long‐term imeglimin therapy was associated with sustained glycemic improvement and early as well as sustained numerical changes in an insulin secretion index without an increase in overall treatment intensity.

## Graphical abstract

In a real‐world setting, 12‐month imeglimin therapy in Japanese patients with type 2 diabetes provided sustained glycemic improvements and enhanced endogenous insulin secretion. Importantly, these durable metabolic benefits were achieved without requiring an increase in overall glucose‐lowering treatment intensity.

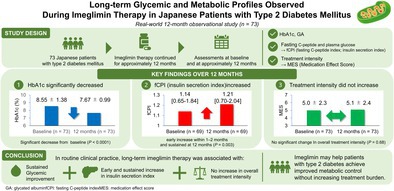

## 1 Introduction

Imeglimin is the first agent of a novel class of oral antidiabetic drugs known as glimins and was approved in Japan in June 2021 for the treatment of type 2 diabetes mellitus (T2DM) [1, 2, 3, 4]. Imeglimin exerts glucose‐lowering effects through multiple pharmacological mechanisms, including enhancement of glucose‐stimulated insulin secretion and improvement of insulin sensitivity, as well as suppression of hepatic glucose production [5, 6]. The efficacy and safety of imeglimin have been validated in pivotal trials covering a broad clinical spectrum, including its use as monotherapy or as an add‐on to existing glucose‐lowering therapies [1, 2, 3, 4].

Hemoglobin A1c (HbA1c) is widely used as the standard marker for assessing long‐term glycemic control, reflecting average blood glucose levels over the preceding three months [7, 8]. However, HbA1c does not fully capture short‐term glycemic variability or postprandial glucose excursions, which may be particularly relevant when evaluating therapies with insulinotropic properties. Glycated albumin (GA), which reflects glycemia over approximately 2–3 weeks and is independent of erythrocyte lifespan, provides complementary information to HbA1c [9]. The GA/HbA1c ratio has been proposed as an indicator reflecting glycemic variability, postprandial hyperglycemia, and, in part, endogenous insulin secretory capacity [10]. Previous studies have suggested that a higher GA/HbA1c ratio is associated with impaired insulin secretion and greater glycemic excursions [10, 11]. Therefore, simultaneous evaluation of HbA1c, GA, and the GA/HbA1c ratio may provide additional insights into the pathophysiological effects of imeglimin.

Although the glucose‐lowering efficacy of imeglimin has been established in randomised controlled trials, data describing long‐term glycemic and metabolic changes during routine clinical practice remain limited [12]. In particular, the longitudinal behaviour of intermediate glycemic markers such as GA, as well as indices reflecting endogenous insulin secretion during prolonged imeglimin therapy, has not been sufficiently characterised [13, 14]. Moreover, how intermediate‐term glycemic markers and insulin secretion indices behave concurrently during prolonged imeglimin therapy in real‐world practice remains unclear. Clarifying these aspects provides deeper insights into the long‐term clinical outcomes of imeglimin therapy in real‐world clinical practice. Accordingly, this study aimed to describe longitudinal changes in glycemic and metabolic parameters during imeglimin therapy in Japanese patients with T2DM in a routine clinical setting.

## 2 Materials and Methods

### 2.1 Study Designs and Patients

This study was designed to characterise long‐term glycemic and metabolic profiles in routine clinical practice. Accordingly, this analysis focused on patients with available 12‐month follow‐up data during continued imeglimin therapy, rather than an intention‐to‐treat population. This study was conducted at Osaka General Medical Center. Adults aged ≥ 20 years with T2DM who initiated oral imeglimin therapy between June 1, 2021, and March 31, 2025, were identified from electronic medical records.

Imeglimin was prescribed at the approved dose of up to 1000 mg twice daily in accordance with routine clinical practice in Japan. Concomitant glucose‐lowering therapies were allowed at the discretion of the treating physicians and were not restricted by the study protocol.

**FIGURE 1:**
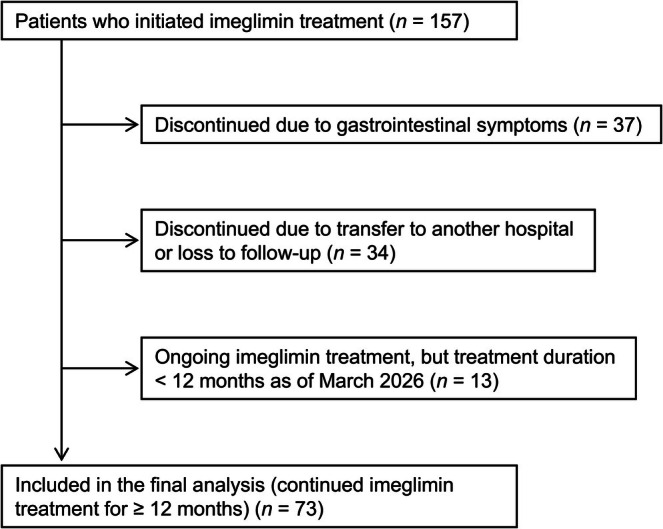
Patient flow diagram A total of 157 patients initiated imeglimin treatment. Patients were excluded if imeglimin was discontinued due to gastrointestinal symptoms (*n* = 37), if all prescriptions were discontinued due to transfer to another hospital or loss to follow‐up (*n* = 34), or if imeglimin treatment was ongoing but the duration of therapy was < 12 months as of March 2026 (*n* = 13). Finally, 73 patients who continued imeglimin therapy for ≥ 12 months were included in the analysis.

Patients were eligible for inclusion if they continued imeglimin treatment for at least 12 months and had sufficient clinical data available for longitudinal evaluation. Exclusion criteria included a diagnosis of type 1 diabetes mellitus, pregnancy or breastfeeding, acute severe infection, pre‐ or postoperative status, or insufficient clinical data for follow‐up analyses. Patients with advanced renal dysfunction requiring discontinuation of imeglimin were also excluded in accordance with the approved prescribing information during the study period. The process of patient selection and exclusion is summarised in Figure 1.

### 2.2 Measurements

Clinical, laboratory, and demographic data were retrieved from electronic medical records at baseline and at follow‐up time points, including 1, 3, 6, and 12 months after initiation of imeglimin, when available. No imputation was performed for missing data due to the exploratory nature and real‐world design. Anthropometric measurements, including height and body weight, were recorded at baseline and during follow‐up visits, and body mass index (BMI) was calculated as body weight (kg) divided by height squared (m^2^). We calculated the estimated glomerular filtration rate (eGFR) using the Chronic Kidney Disease Epidemiology Collaboration (CKD‐EPI) equation, modified for the Japanese population [15]. To evaluate the relationship between short‐term and long‐term glycemic indices, we determined the GA/HbA1c ratio [10]. For parameters requiring strict fasting conditions (fasting plasma glucose and fasting C‐peptide), data were extracted only from visits occurring between 10 and 14 months after initiation. This approach was adopted to ensure clinical reliability. Blood samples were generally collected in the morning after an overnight fast; however, fasting duration and sampling intervals were not strictly standardised owing to the retrospective study design. The fasting C‐peptide index (fCPI) was calculated as [fasting serum C‐peptide (ng/mL) / fasting plasma glucose (mg/dL)] × 100 [16]. To evaluate early changes in beta‐cell function, fCPI measurements obtained within 1–2 months after initiation of imeglimin were additionally analyzed in patients with available paired data. As early‐phase measurements were not available for all patients, this analysis was conducted in a subset of the study population. When multiple measurements were available within this period, the earliest value was used for analysis.

To quantitatively assess changes in the overall intensity of glucose‐lowering therapy, the medication effect score (MES) was calculated according to a previously validated method [17]. For each antidiabetic agent, the MES was calculated as the ratio of the prescribed daily dose to the maximum approved daily dose, weighted by a drug‐specific adjustment factor that reflects the agent's estimated maximum HbA1c‐lowering efficacy. For insulin therapy, the MES was calculated by normalizing the total daily dose to body weight (units/kg/day) and applying a predefined adjustment factor. For glucagon‐like peptide‐1 (GLP‐1) receptor agonists, the MES was calculated according to the previously validated drug‐specific weighting factors used for injectable glucose‐lowering agents. Given the absence of a validated MES for glucose‐dependent insulinotropic polypeptide (GIP) /GLP‐1 receptor agonist, tirzepatide, an exploratory value of 1.8 was assigned to this agent, exclusively for use in sensitivity analyses. The primary interpretation of treatment intensity was not dependent on this assumption. The total MES was calculated as the sum of all individual drug‐specific MES values and was assessed at baseline (imeglimin initiation) and at 12 months. In the present analysis, imeglimin was not included in the MES calculation, and therefore MES reflects the intensity of concomitant glucose‐lowering therapies excluding imeglimin. In addition, changes in MES were evaluated to assess the intensity of background therapies independently of imeglimin.

### 2.3 Definition of Dose Maintenance

Imeglimin dose maintenance was evaluated in an exploratory analysis among patients who continued imeglimin treatment for at least 12 months. Patients were classified as the dose‐maintenance group if they maintained the full approved dose of imeglimin (2000 mg/day; 1000 mg twice daily) from treatment initiation through 12 months, as indicated by the treatment adherence indicator recorded in the electronic medical records. Patients who started imeglimin at a reduced dose, required dose reduction, or did not maintain the full dose during follow‐up were categorised as the non‐maintenance group.

### 2.4 Outcomes

The primary outcome was the observed change in HbA1c from baseline to approximately 12 months during imeglimin therapy (ΔHbA1c). Secondary outcomes included changes in GA, the GA/HbA1c ratio, and the fCPI over the same period. We also evaluated changes in liver enzymes and renal function parameters to assess the long‐term metabolic effects and safety of sustained imeglimin treatment.

### 2.5 Statistical Analysis

Continuous variables are presented as mean ± standard deviation (SD) or median [interquartile range], as appropriate. Paired comparisons were conducted using parametric or non‐parametric tests, as appropriate. A two‐sided *p* value < 0.05 was considered statistically significant. Comparisons between patients who continued and those who discontinued imeglimin treatment were conducted using unpaired *t*‐tests, as appropriate. Longitudinal changes in HbA1c over the 12 months were analyzed using repeated‐measures analysis of variance. For multiple comparisons across different time points, Steel's test was applied.

Concomitant glucose‐lowering medications at baseline and after 12 months were summarised descriptively. Changes in concomitant oral antidiabetic drugs (OADs) and insulin doses were recorded. To adjust for changes in concomitant treatment, patients were stratified by their non‐imeglimin medication status: intensified, reduced, or stable. Exploratory subgroup analyses stratified by dose maintenance were performed among patients who continued imeglimin treatment for at least 12 months. In addition, individual changes (Δ) in the fCPI and the GA/HbA1c ratio from baseline to 12 months were calculated and summarised descriptively for graphical presentation, and their relationship was explored using Spearman's correlation analysis. We performed exploratory analyses to evaluate the baseline clinical characteristics associated with the continuation or discontinuation of imeglimin in routine clinical practice. Furthermore, a sensitivity analysis was performed by excluding patients who initiated other insulin secretagogues during the follow‐up period to confirm the independent effects of imeglimin. All statistical analyses were performed using JMP software, version 19 (SAS Institute Inc., Cary, NC, USA).

## 3 Results

### 3.1 Patient Characteristics

Figure 1 shows the flow of patients enrolled in the present study. Of the 157 patients who initiated imeglimin, 73 who continued treatment for at least 12 months were included in the final analysis.

Baseline clinical characteristics of the study population are summarised in Table 1. The mean age of the patients was 61.5 ± 13.7 years, and the mean duration of T2DM was 16 [6.5–22] years. Male patients accounted for 60.3% (*n* = 44) of the study population. The mean BMI was 24.7 ± 4.3 kg/m^2^. At the time of imeglimin initiation, glycemic control was suboptimal, as evidenced by a mean baseline HbA1c of 8.55% ± 1.38% and a median GA of 20.2 [17.0–23.6]%. The baseline GA/HbA1c ratio was 2.41 [2.18–2.73] (*n* = 72). The baseline fCPI was 1.14 [0.65–1.84] (*n* = 69), reflecting modest endogenous insulin secretory capacity. Most patients were receiving treatment with multiple hypoglycemic agents in addition to imeglimin. At baseline, patients were receiving an average of 2.40 ± 1.30 antidiabetic agents, which remained largely stable at 2.52 ± 1.17 after 12 months of therapy. Baseline renal function and liver enzyme levels were within clinically acceptable ranges.

**TABLE 1:** Baseline characteristics of the study population.

Characteristic	Value
Number of patients	73
Duration of T2DM (years)	16 [6.5–22]
Age (years)	61.5 ± 13.7
Male sex, *n* (%)	44 (60.3)
Height (cm)	163.5 ± 9.7
Body weight (kg)	66.3 ± 14.1
BMI (kg/m^2^)	24.7 ± 4.3
HbA1c (%)	8.55 ± 1.38
GA (%)	20.2 [17.0–23.6]
GA/HbA1c ratio	2.41 [2.18–2.73]
Fasting plasma glucose (mg/dL)	170 [138–213]
Serum creatinine (mg/dL)	0.88 ± 0.38
eGFR (mL/min/1.73 m^2^)	70.9 ± 23.0
Fasting serum C‐peptide (ng/mL)	2.14 [1.28–3.09]
fCPI	1.14 [0.65–1.84]

*Note:* Data are presented as mean ± SD or number (%) as appropriate. We analyzed baseline data from the 73 patients who completed at least 12 months of imeglimin therapy.

Abbreviations: BMI, body mass index; eGFR, estimated glomerular filtration rate; fCPI, fasting C‐peptide index; GA, glycated albumin; HbA1c, hemoglobin A1c; T2DM, type 2 diabetes mellitus.

Regarding changes in concomitant glucose‐lowering therapies over the 12 months, 25 patients (34.2%) continued their baseline medications without any modifications, and 7 patients (9.6%) had their background medications strictly reduced. The remaining 41 patients (56.2%) underwent some form of medication adjustment, including additions, discontinuations, or dose changes as part of routine clinical care. When quantifying these adjustments using the MES, excluding imeglimin from the calculation, the net change in MES (ΔMES) was zero in 32 patients (43.8%), positive in 30 patients (41.1%), and negative in 11 patients (15.1%). Overall, the MES for the entire cohort did not change significantly from baseline to 12 months (Wilcoxon signed‐rank test, *p* = 0.115).

### 3.2 Primary Outcome

**FIGURE 2:**
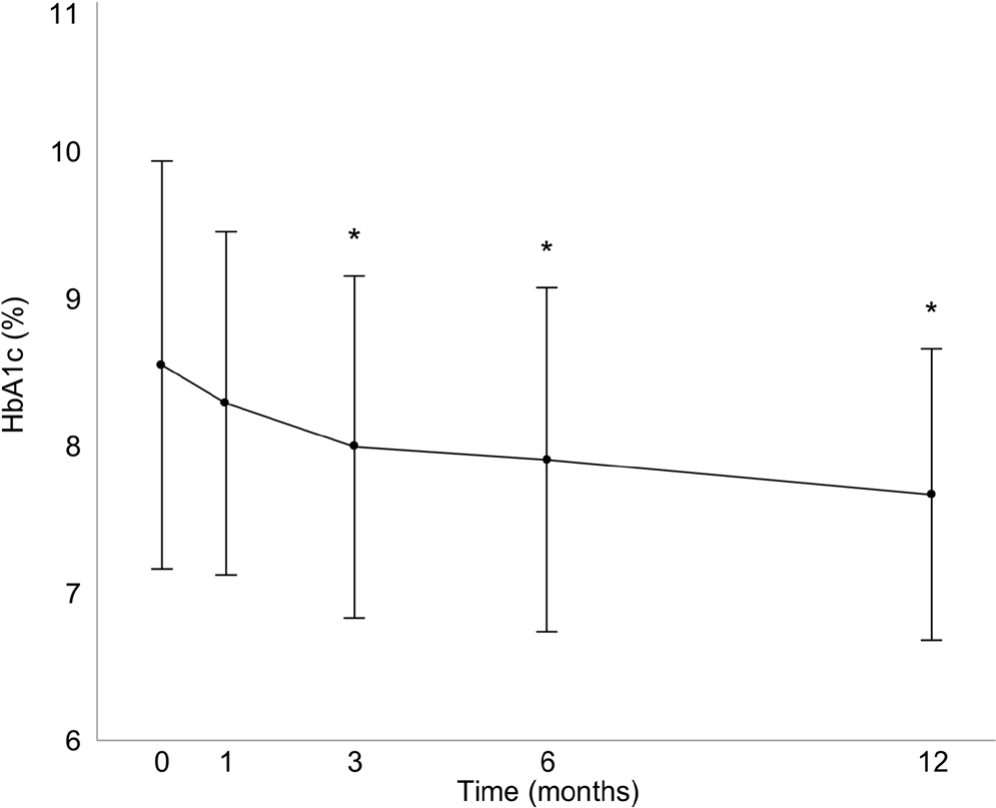
Longitudinal changes in hemoglobin A1c (HbA1c) during imeglimin treatment. Data are presented as mean ± standard deviation. Overall differences over time were assessed using repeated‐measures analysis of variance. *p < 0.05 versus baseline by Steel's test.

HbA1c showed a significant overall change over time during the 12 months after initiation of imeglimin treatment (repeated‐measures analysis of variance, *p* < 0.0001; Figure 2). HbA1c did not show a significant reduction in 1 month but demonstrated significant decreases from 3 months onward.

### 3.3 Changes in Metabolic Parameters

Changes in glycemic and metabolic parameters from baseline to 12 months are summarised in Table 2. Mean GA values (*n* = 72) were 20.2 [17.0–23.6] % at baseline, 19.4 [16.6–22.4]% at 1 month, 18.9 [15.7–22.6]% at 3 months, 19.1 [16.1–22.3]% at 6 months, and 18.4 [15.5–21.9]% at 12 months. According to the Wilcoxon signed‐rank test, GA levels at all follow‐up time points (1, 3, 6, and 12 months) showed significant reductions compared with the baseline (all *p* < 0.05). However, when adjusted for multiple comparisons using Steel's test, a significant difference relative to the baseline was observed only at 12 months (*p* = 0.036). By contrast, the GA/HbA1c ratio (*n* = 72) remained stable throughout the study period, with values of 2.41 [2.18–2.73] at baseline, 2.39 [2.08–2.65] at 1 month, 2.39 [2.14–2.70] at 3 months, 2.41 [2.12–2.70] at 6 months, and 2.38 [2.17–2.69] at 12 months. In the Wilcoxon signed‐rank test, a significant difference compared with the baseline was observed only at 1 month (*p* = 0.015); however, no significant differences were found at any time point when analyzed using Steel's test for multiple comparisons. Both HbA1c and GA decreased significantly during follow‐up, whereas the GA/HbA1c ratio remained stable. This pattern suggests that overall glycemic exposure improved without a disproportionate change in short‐term glycemic dynamics. Fasting serum C‐peptide levels showed no significant change over 12 months; however, the fCPI exhibited a statistically significant improvement from baseline to 12 months. The median fCPI increased from 1.14 [0.65–1.84] at baseline to 1.21 [0.79–2.01] at 12 months (*n* = 69, Wilcoxon signed‐rank test, *p* = 0.003). This finding indicates an overall upward shift in a surrogate index of endogenous insulin secretory capacity. Although the median GA/HbA1c ratio remained unchanged, substantial inter‐individual variability was observed. An increase in fCPI was observed in most patients, while a substantial proportion showed stable or decreased values. Early‐phase fCPI (1–2 months) was available for paired analysis in 15 patients. The median fCPI increased from 0.89 [0.56–1.47] at baseline to 1.09 [0.87–1.84] at 1–2 months (Wilcoxon signed‐rank test, *p* = 0.035), indicating an early improvement in an index of endogenous insulin secretion. No clinically relevant deterioration in liver or renal function was observed during long‐term imeglimin treatment.

**TABLE 2:** Changes in clinical parameters before and after imeglimin treatment.

Parameter	Baseline	After treatment	*p*
HbA1c (%)	8.55 ± 1.38	7.67 ± 0.99	< 0.0001
Fasting plasma glucose (mg/dL)	170 [138–213]	148 [131–177]	0.006
GA (%)	20.2 [17.0–23.6]	18.4 [15.5–21.9]	< 0.0001
GA/HbA1c ratio	2.41 [2.18–2.73]	2.38 [2.17–2.69]	0.253
Fasting serum C‐peptide (ng/mL)	2.14 [1.28–3.09]	1.87 [1.22–3.36]	0.325
fCPI	1.14 [0.65–1.84]	1.21 [0.79–2.01]	0.003
MES	1.17 [0.61–1.84]	1.29 [0.68–1.80]	0.115

*Note:* Data are presented as mean ± standard deviation or median [interquartile range], as appropriate. Changes from baseline to 12 months were analyzed using paired *t*‐tests or the Wilcoxon signed‐rank test.

Abbreviations: fCPI, fasting C‐peptide index; GA, glycated albumin; HbA1c, hemoglobin A1c; MES, medication effect score.

Concomitant glucose‐lowering medications at baseline and after 12 months are summarised in Table 3. During the follow‐up period, adjustments to these concomitant therapies were made in 48 of the 73 patients (65.8%). While the proportion of patients receiving GLP‐1 receptor agonists decreased over 12 months, the use of biguanides and SGLT2 inhibitors increased modestly. Four patients were receiving the dual GIP/GLP‐1 receptor agonist tirzepatide, which was distinguished from selective GLP‐1 receptor agonists in the analysis.

**TABLE 3:** Concomitant glucose‐lowering medications at imeglimin initiation and after 12 months.

Medication	Baseline *n* (%)	12 months *n* (%)
Insulin	24 (32.9%)	25 (34.2%)
GLP‐1 receptor agonist	23 (31.5%)	19 (26.0%)
GIP/GLP‐1 receptor agonist	0 (0%)	4 (5.5%)
Sulfonylurea	19 (26.0%)	20 (27.4%)
SGLT2 inhibitor	37 (50.7%)	40 (54.8%)
DPP‐4 inhibitor	27 (37.0%)	28 (38.4%)
Biguanide (metformin)	40 (54.8%)	44 (60.3%)
Glinide	3 (4.1%)	2 (2.7%)
Thiazolidinedione	2 (2.7%)	2 (2.7%)
Alpha‐glucosidase inhibitor	0 (0%)	0 (0%)

*Note:* Data are expressed as number (%). During the 12‐month follow‐up, concomitant glucose‐lowering medications were adjusted in 48 patients (65.8%).

Abbreviations: DPP‐4, dipeptidyl peptidase‐4; GIP, glucose‐dependent insulinotropic polypeptide; GLP‐1, glucagon‐like peptide‐1; SGLT2, sodium–glucose cotransporter 2.

**FIGURE 3:**
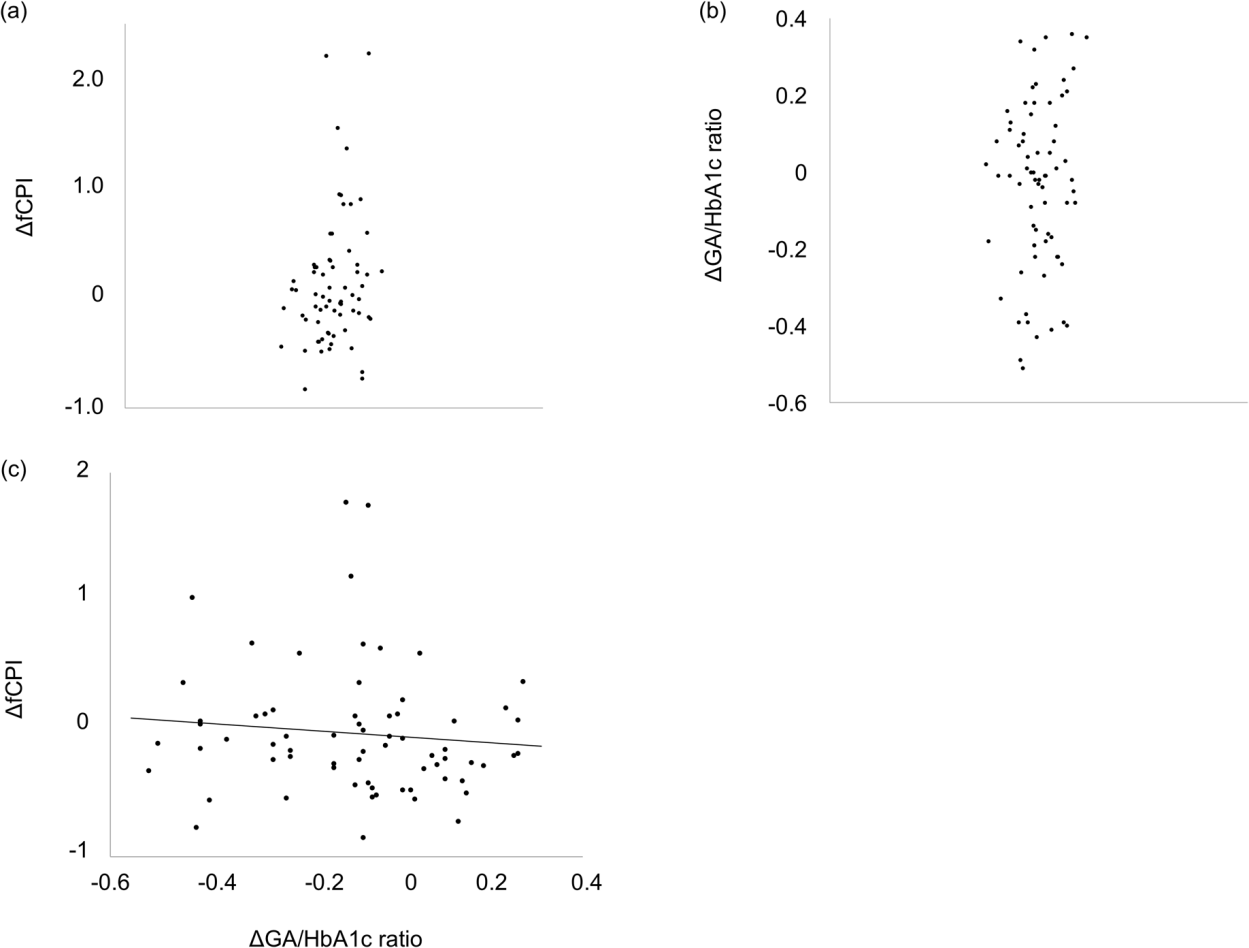
Individual changes in metabolic parameters from baseline to 12 months. (a) Changes in the C‐peptide index (ΔfCPI) (*n* = 69) and (b) changes in the glycated albumin‐to‐hemoglobin A1c ratio (ΔGA/HbA1c) (*n* = 72). (c) Relationship between ΔfCPI and ΔGA/HbA1c ratio. Spearman's correlation analysis showed no significant association between ΔfCPI and ΔGA/HbA1c ratio (*n* = 69, *ρ* = −0.14, *p* = 0.251). Fasting blood samples for fCPI were specifically extracted from a 10‐ to 14‐month window to ensure accuracy, while other parameters were assessed at approximately 12 months. fCPI, fasting C‐peptide index; GA, glycated albumin; HbA1c, hemoglobin A1c.

The individual changes (Δ) in metabolic parameters from baseline to 12 months are shown in Figure 3. Regarding endogenous insulin secretory capacity, the distribution of the change in the fasting C‐peptide index (ΔfCPI) showed a significant upward shift from zero (*n* = 69, 0.10 [−0.11–0.36]; *p* = 0.003 by Wilcoxon signed‐rank test; Figure 3a), reflecting an overall improvement in insulin secretory capacity across the cohort. Even after excluding the four patients who initiated or switched to tirzepatide during the study period, the increase in fCPI remained highly significant (*n* = 65, Baseline vs. 12 months: *p* = 0.001, Wilcoxon signed‐rank test). In contrast, the change in the GA/HbA1c ratio (ΔGA/HbA1c) was not significantly different from zero (*n* = 72, −0.01 [−0.19–0.12]; *p* = 0.253; Figure 3b), suggesting that the balance between short‐term and long‐term glycemic control remained stable during the 12‐month follow‐up. Furthermore, we examined the relationship between these changes. Spearman's correlation analysis revealed no significant association between ΔfCPI and ΔGA/HbA1c ratio (*n* = 69, *ρ* = −0.14, *p* = 0.251; Figure 3c). These findings suggest that these parameters may reflect different physiological aspects of glucose metabolism; however, this should not be overinterpreted.

### 3.4 Exploratory Analysis of Imeglimin Dose Maintenance

Among patients who remained on imeglimin for 12 months, 46 remained on the full dose, whereas 27 underwent dose reductions. No significant differences were observed in changes in HbA1c, GA, or fCPI over 12 months when stratified by dose maintenance.

### 3.5 Baseline Characteristics Associated With Discontinuation of Imeglimin

Baseline clinical characteristics were compared between patients who continued imeglimin treatment for at least 12 months and those who discontinued treatment during follow‐up. The discontinuation group exhibited more complex baseline treatment regimens, characterised by a higher prevalence of insulin therapy and greater metformin doses than the continuation group. Patients in the discontinuation group demonstrated worse baseline glycemic control, whereas age, T2DM duration, and BMI did not differ significantly between the two groups.

### 3.6 Sensitivity Analysis Excluding Patients With New Insulin Secretagogues

To confirm the robustness of the primary findings, a sensitivity analysis was performed by excluding nine patients who initiated or intensified other potent glucose‐lowering therapies during the follow‐up period. Specifically, we excluded one patient each for the initiation of a sulfonylurea, a combination of a sulfonylurea and a DPP‐4 inhibitor, a DPP‐4 inhibitor, a GLP‐1 receptor agonist, and insulin therapy, as well as four patients who switched from a GLP‐1 receptor agonist to a dual GIP/GLP‐1 receptor agonist. In the remaining 64 patients, the improvements in metabolic parameters remained highly significant; HbA1c decreased from 8.50% ± 1.36% at baseline to 7.68% ± 1.01% at 12 months (*p* < 0.0001), and fCPI increased from 1.25 [0.69–1.93] to 1.26 [0.82–2.14] (*p* = 0.006). These results reinforce the independent contribution of imeglimin to the observed clinical benefits.

## 4 Discussion

We assessed the 12‐month longitudinal effects of imeglimin on glycemic and metabolic parameters in a real‐world cohort of Japanese patients with T2DM. The principal observations included sustained improvements in glycemic control, a statistically significant increase in the fCPI, and the maintenance of overall treatment intensity, despite frequent adjustments to background therapy. Such real‐world observational analyses are increasingly recognised as important for characterizing the longitudinal metabolic effects of glucose‐lowering therapies under routine clinical practice conditions [18].

Significant and sustained reductions in HbA1c and GA were observed throughout the 12‐month follow‐up period. Importantly, GA decreased from the early phase after imeglimin initiation, as reflected by consistent reductions at 1, 3, and 6 months. Despite this early improvement in GA, the GA/HbA1c ratio remained relatively stable over time. This interpretation is supported by the evaluation of the GA/HbA1c ratio at multiple time points, including 1, 3, 6, and 12 months, which showed only a transient change at 1 month without sustained differences thereafter. This suggests that improvements in glycemic control were not accompanied by a disproportionate change between short‐ and long‐term glycemic indices. Taking together, this pattern suggests that imeglimin improves overall glycemic control from the early phase without markedly altering the balance between short‐term and long‐term glycemic markers. Therefore, the observed improvement may reflect a relatively uniform reduction in blood glucose levels rather than a selective effect on glycemic variability or postprandial excursions. Interpretation of glycemic control during therapeutic changes can be challenging, as HbA1c alone may not fully capture short‐term or differential metabolic responses [11]. Baseline GA values appeared relatively lower than expected based on HbA1c; however, the underlying reasons could not be fully evaluated in this retrospective study. These results are consistent with findings from pivotal clinical trials and previous real‐world studies, supporting the durability of imeglimin's glucose‐lowering effect under routine clinical practice conditions [1, 3, 4, 18].

Although the absolute increase in fasting C‐peptide index was modest, a marked increase was observed in the early phase (1–2 months), suggesting a potential early effect of imeglimin on beta‐cell function. Notably, this early increase in fCPI preceded the improvement in HbA1c, which became apparent from 3 months onward, and occurred in parallel with early changes in GA [19]. Such early dynamics may reflect improved beta‐cell responsiveness shortly after treatment initiation. A significant increase in fCPI was also observed over 12 months, indicating a sustained numerical improvement in an index of endogenous insulin secretion [20, 21]. Assessment of endogenous insulin secretion provides important pathophysiological information in patients with T2DM, particularly when interpreting the metabolic effects of agents with potential insulinotropic properties [21, 22].

The fCPI is a validated surrogate marker of endogenous insulin secretory capacity in patients with T2DM [16, 23], and its improvement is biologically plausible based on previously reported insulinotropic properties of imeglimin. Imeglimin has been shown to enhance glucose‐stimulated insulin secretion through improvement of mitochondrial bioenergetics and preservation of beta‐cell function [24, 25]. Furthermore, morphological improvements in mitochondria and an increased number of insulin granules in pancreatic β‐cells have been observed following imeglimin treatment [26].

However, as this was an observational study, a definitive causal relationship between imeglimin therapy and improved β‐cell function could not be established. The lack of a significant correlation between ΔfCPI and ΔGA/HbA1c suggests these parameters may reflect different physiological aspects of glucose metabolism. This observation may reflect the multifactorial nature of glycemic regulation during imeglimin therapy, including effects on insulin secretion as well as insulin sensitivity or suppression of hepatic glucose production, in line with its proposed dual mechanism of action [10, 20]. However, these findings should be interpreted with caution and do not establish distinct mechanistic pathways.

While background therapy was adjusted in most patients (65.8%), the overall treatment intensity—as assessed using the MES with imeglimin excluded—remained unchanged at 12 months. This suggests that the overall intensity of concomitant glucose‐lowering therapy did not substantially increase, although potential contributions from treatment adjustments cannot be excluded. However, treatment modifications were frequent, and therefore the possibility that changes in background therapies (e.g., increased use of SGLT2 inhibitors or metformin) contributed to the observed improvements cannot be completely excluded [17]. Thus, the observed glycemic improvements may reflect both the direct effects of imeglimin and changes in background therapies in routine clinical practice.

These findings should be interpreted within the context of real‐world clinical practice, where multiple treatment adjustments often occur in parallel.

This apparent dissociation between frequent medication adjustments and stable MES highlights an important methodological and clinical consideration. Simple assessments of drug counts or types, as presented in Table 3, can lead to an overestimation of treatment escalation, failing to reflect actual treatment intensity [27]. In contrast, MES captures cumulative pharmacologic burden by accounting for drug potency and dosing intensity [17]. In this context, the stability of MES may reflect both the effects of imeglimin and changes in background therapy. Consistent findings were observed even after excluding patients who initiated additional glucose‐lowering therapies during follow‐up. Although an exploratory MES value was assigned to tirzepatide due to the absence of a validated definition for this agent, the primary conclusions regarding treatment intensity were not dependent on this assumption.

Regarding safety, 23.6% of patients in the initial cohort discontinued imeglimin because of gastrointestinal symptoms. This rate slightly exceeded clinical trial findings [3, 4]. This difference may reflect the frequent concurrent use of metformin, which shares a similar gastrointestinal safety profile [28]. These findings underscore the importance of careful monitoring and individualised dose titration when initiating imeglimin in patients receiving complex background regimens. In addition, fasting status and follow‐up timing were not strictly standardised, which should be considered when interpreting changes in insulin secretion indices.

Taken together, these findings suggest that imeglimin therapy is associated with early and sustained improvements in glycemic control, accompanied by a numerical early enhancement of an insulin secretion index, without a clear increase in overall treatment intensity under real‐world clinical conditions.

Therefore, we interpret the observed improvements in glycemic control and fasting C‐peptide index as outcomes achievable in patients who tolerate and continue long‐term imeglimin therapy, rather than effects generalisable to all patients who initiate treatment.

## 5 Limitations

Several limitations of this study warrant consideration. First, the retrospective, single‐center nature of this study and the absence of a control group preclude definitive causal inferences. Second, the longitudinal analysis inherently carries a survivor bias, as it focuses exclusively on patients who successfully maintained imeglimin therapy for 12 months, potentially excluding those who discontinued treatment due to early adverse effects or insufficient efficacy. Furthermore, while the MES remained stable, the dynamic adjustments of concomitant glucose‐lowering therapies in a real‐world setting may still confound the isolation of imeglimin‐specific effects. In addition, early‐phase fCPI measurements were available only in a subset of patients, which may introduce selection bias in the interpretation of short‐term changes. Despite these constraints, the 12‐month follow‐up and the granular evaluation of C‐peptide dynamics offer clinically significant insights into the metabolic trajectory of patients treated with imeglimin in routine clinical practice.

## 6 Conclusions

This real‐world study confirms that long‐term imeglimin therapy was associated with sustained improvements in glycemic profiles and numerical changes in an index of endogenous insulin secretion without requiring an increase in overall treatment intensity. These findings support imeglimin as a therapeutic option that may facilitate metabolic improvement while maintaining treatment simplicity in patients with T2DM under routine clinical care conditions.

## Author Contributions

**Shuji Suganami:** resources, investigation, formal analysis. **Yohei Fujita:** conceptualization, methodology, data curation, investigation, validation, formal analysis, writing – original draft, writing – review and editing, visualization, project administration, resources, software. **Toshio Matsui:** resources, investigation, data curation. **Masahisa Hata:** resources, investigation, validation. **Masahiro Hatazaki:** supervision, resources. **Yuka Morita:** resources, investigation, data curation.

## Ethics Statement

All procedures performed in this study were in accordance with the ethical standards of the Ethics Committee of Osaka General Medical Center (Approval No. 2024‐050, October 28, 2024) and with the Declaration of Helsinki and its later amendments.

## Consent

In accordance with ethical guidelines for retrospective chart reviews, informed consent was not required, as all patient information was fully anonymised prior to analysis. Patients were informed about the study and were provided the opportunity to opt out.

## Conflicts of Interest

The authors declare no conflicts of interest.

## Data Availability

The datasets are available from the corresponding author on reasonable request.

## References

[1] J. Dubourg , P. Fouqueray , C. Thang , J. M. Grouin , and K. Ueki , “Efficacy and Safety of Imeglimin Monotherapy Versus Placebo in Japanese Patients With Type 2 Diabetes (TIMES 1): A Double‐Blind, Randomized, Placebo‐Controlled, Parallel‐Group, Multicenter Phase 3 Trial,” Diabetes Care 44 (2021): 952–959. 33574125 10.2337/dc20-0763

[2] J. Dubourg , K. Ueki , J. M. Grouin , and P. Fouqueray , “Efficacy and Safety of Imeglimin in Japanese Patients With Type 2 Diabetes: A 24‐Week, Randomized, Double‐Blind, Placebo‐Controlled, Dose‐Ranging Phase 2b Trial,” Diabetes, Obesity and Metabolism 23 (2021): 800–810. 10.1111/dom.14285 PMC7898540 33275318

[3] J. Dubourg , P. Fouqueray , D. Quinslot , J. M. Grouin , and K. Kaku , “Long‐Term Safety and Efficacy of Imeglimin as Monotherapy or in Combination With Existing Antidiabetic Agents in Japanese Patients With Type 2 Diabetes (TIMES 2): A 52‐Week, Open‐Label, Multicentre Phase 3 Trial,” Diabetes, Obesity and Metabolism 24 (2022): 609–619. 10.1111/dom.14613 PMC9305103 34866306

[4] C. Reilhac , J. Dubourg , C. Thang , J. M. Grouin , P. Fouqueray , and H. Watada , “Efficacy and Safety of Imeglimin Add‐On to Insulin Monotherapy in Japanese Patients With Type 2 Diabetes (TIMES 3): A Randomized, Double‐Blind, Placebo‐Controlled Phase 3 Trial With a 36‐Week Open‐Label Extension Period,” Diabetes, Obesity and Metabolism 24 (2022): 838–848. 10.1111/dom.14642 PMC9302620 34984815

[5] G. Vial , F. Lamarche , C. Cottet‐Rousselle , S. Hallakou‐Bozec , A. L. Borel , and E. Fontaine , “The Mechanism by Which Imeglimin Inhibits Gluconeogenesis in Rat Liver Cells,” Endocrinology, Diabetes & Metabolism 4 (2021): e00211. 10.1002/edm2.211 PMC8029524 33855213

[6] P. Theurey , C. Thang , V. Pirags , et al., “Phase 2 Trial With Imeglimin in Patients With Type 2 Diabetes Indicates Effects on Insulin Secretion and Sensitivity,” Endocrinology, Diabetes & Metabolism 5 (2022): e371. 10.1002/edm2.371 PMC9659655 36239048

[7] S. I. Sherwani , H. A. Khan , A. Ekhzaimy , A. Masood , and M. K. Sakharkar , “Significance of HbA1c Test in Diagnosis and Prognosis of Diabetic Patients,” Biomarker Insights 11 (2016): 95–104. 27398023 10.4137/BMI.S38440 PMC4933534

[8] American Diabetes Association Professional Practice Committee , “6. Glycemic Goals and Hypoglycemia: Standards of Care in Diabetes‐2025,” Diabetes Care 48 (2025): S128–S145. 39651981 10.2337/dc25-S006 PMC11635034

[9] M. Koga and S. Kasayama , “Clinical Impact of Glycated Albumin as Another Glycemic Control Marker,” Endocrine Journal 57 (2010): 751–762. 20724796 10.1507/endocrj.k10e-138

[10] M. Koga , J. Murai , H. Saito , and S. Kasayama , “Glycated Albumin and Glycated Hemoglobin Are Influenced Differently by Endogenous Insulin Secretion in Patients With Type 2 Diabetes,” Diabetes Care 33 (2010): 270–272. 19846794 10.2337/dc09-1002 PMC2809261

[11] S. Takahashi , H. Uchino , T. Shimizu , et al., “Comparison of Glycated Albumin (GA) and Glycated Hemoglobin (HbA1c) in Type 2 Diabetic Patients: Usefulness of GA for Evaluation of Short‐Term Changes in Glycemic Control,” Endocrine Journal 54 (2007): 139–144. 17159300 10.1507/endocrj.k06-103

[12] K. Hagi , M. Nitta , H. Watada , K. Kaku , and K. Ueki , “Efficacy, Safety and Tolerability of Imeglimin in Patients With Type 2 Diabetes Mellitus: A Meta‐Analysis of Randomized Controlled Trials,” Journal of Diabetes Investigation 14 (2023): 1246–1261. 37610062 10.1111/jdi.14070 PMC10583642

[13] M. Fauzi , T. Murakami , D. Yabe , and N. Inagaki , “Current Understanding of Imeglimin Action on Pancreatic β‐Cells: Involvement of Mitochondria and Endoplasmic Reticulum Homeostasis,” Journal of Diabetes Investigation 14 (2023): 186–188. 36453164 10.1111/jdi.13951 PMC9889698

[14] H. Yanai , H. Adachi , M. Hakoshima , and H. Katsuyama , “Glucose‐Lowering Effects of Imeglimin and Its Possible Beneficial Effects on Diabetic Complications,” Biology (Basel) 12 (2023): 726. 37237539 10.3390/biology12050726 PMC10215333

[15] M. Horio , E. Imai , Y. Yasuda , T. Watanabe , and S. Matsuo , “Modification of the CKD Epidemiology Collaboration (CKD‐EPI) Equation for Japanese: Accuracy and Use for Population Estimates,” American Journal of Kidney Diseases 56 (2010): 32–38. 20416999 10.1053/j.ajkd.2010.02.344

[16] S. Funakoshi , S. Fujimoto , A. Hamasaki , et al., “Utility of Indices Using C‐Peptide Levels for Indication of Insulin Therapy to Achieve Good Glycemic Control in Japanese Patients With Type 2 Diabetes,” Journal of Diabetes Investigation 2 (2011): 297–303. 24843502 10.1111/j.2040-1124.2010.00096.x PMC4014971

[17] A. S. Alexopoulos , W. S. Yancy , D. Edelman , et al., “Clinical Associations of an Updated Medication Effect Score for Measuring Diabetes Treatment Intensity,” Chronic Illness 17 (2021): 451–462. 31653175 10.1177/1742395319884096 PMC7182482

[18] H. Katsuyama , M. Hakoshima , T. Heshiki , S. Iida , H. Adachi , and H. Yanai , “Real‐World Effectiveness of Imeglimin in Patients With Type 2 Diabetes: A Retrospective Longitudinal Study in Japan,” Diabetes Research and Clinical Practice 213 (2024): 111752. 38908549 10.1016/j.diabres.2024.111752

[19] Q. Yingyue , K. Sugawara , H. Takahashi , et al., “Stimulatory Effect of Imeglimin on Incretin Secretion,” Journal of Diabetes Investigation 14 (2023): 746–755. 36977210 10.1111/jdi.14001 PMC10204172

[20] S. Hallakou‐Bozec , M. Kergoat , D. E. Moller , and S. Bolze , “Imeglimin Preserves Islet β‐Cell Mass in Type 2 Diabetic ZDF Rats,” Endocrinology, Diabetes & Metabolism 4 (2020): e00193. 10.1002/edm2.193 PMC8029531 33855202

[21] T. Imada , S. Sasaki , H. Yamaguchi , et al., “Imeglimin, Unlike Metformin, Does Not Perturb Differentiation of Human Induced Pluripotent Stem Cells Towards Pancreatic β‐Like Cells and Rather Enhances Gain in β Cell Identity Gene Sets,” Journal of Diabetes Investigation 16 (2025): 584–597. 39829307 10.1111/jdi.14410 PMC11970301

[22] B. Ahrén and G. Pacini , “Importance of Quantifying Insulin Secretion in Relation to Insulin Sensitivity to Accurately Assess Beta Cell Function in Clinical Studies,” European Journal of Endocrinology 150 (2004): 97–104. 14763905 10.1530/eje.0.1500097

[23] T. Tajima , H. Kaga , N. Ito , et al., “Dual Action of Imeglimin on Insulin Secretion and Sensitivity in Type 2 Diabetes,” Diabetes, Obesity and Metabolism 28 (2026): 2744–2756. 10.1111/dom.70449 PMC12992193 41491607

[24] A. G. Jones and A. T. Hattersley , “The Clinical Utility of C‐Peptide Measurement in the Care of Patients With Diabetes,” Diabetic Medicine 30, no. 7 (2013): 803–817. 23413806 10.1111/dme.12159 PMC3748788

[25] S. Hallakou‐Bozec , M. Kergoat , P. Fouqueray , S. Bolze , and D. E. Moller , “Imeglimin Amplifies Glucose‐Stimulated Insulin Release From Diabetic Islets via a Distinct Mechanism of Action,” PLoS One 16 (2021): e0241651. 33606677 10.1371/journal.pone.0241651 PMC7894908

[26] J. Sanada , A. Obata , Y. Fushimi , et al., “Imeglimin Exerts Favorable Effects on Pancreatic β‐Cells by Improving Morphology in Mitochondria and Increasing the Number of Insulin Granules,” Scientific Reports 12 (2022): 13220. 35918386 10.1038/s41598-022-17657-3 PMC9345869

[27] J. George , Y. T. Phun , M. J. Bailey , D. C. Kong , and K. Stewart , “Development and Validation of the Medication Regimen Complexity Index,” Annals of Pharmacotherapy 38 (2004): 1369–1376. 15266038 10.1345/aph.1D479

[28] J. Ito , K. Hagi , K. Kochi , K. Ueki , H. Watada , and K. Kaku , “Gastrointestinal Symptoms in Patients Receiving Imeglimin in Combination With Metformin: A Post‐Hoc Analysis of Imeglimin Clinical Trial Data,” Journal of Diabetes Investigation 16 (2025): 629–638. 39723797 10.1111/jdi.14396 PMC11970294

